# Oxidant/antioxidant state in tissue of prymary and recurrent pterygium

**DOI:** 10.1186/1471-2415-14-149

**Published:** 2014-11-27

**Authors:** Alexandre Kormanovski, Fidelina Parra, Adriana Jarillo-Luna, Eleazar Lara-Padilla, Judith Pacheco-Yépez, Rafael Campos-Rodriguez

**Affiliations:** Section of Postgrade and Investigation, Superior Medicine School, National Polytechnic Institute, Hopelchen Mn316 Lt2, Col. Heroes de Padierna, Del. Tlalpan, México City, DF CP14200 Mexico; Hospital Nuestra Señora de la Luz, Mexico City, Mexico

**Keywords:** Pterygium, Oxidative stress, Antioxidant, Nitric oxide, Gender differences

## Abstract

**Background:**

Pterygium is a disorder of the ocular surface induced by chronic exposure to UV-light. Abundant data is available from patients with primary pterygium, but scarce from those with recurrent pterygium. The present study aimed to explore the oxidant/antioxidant status in tissue of primary and recurrent pterigium in men and women.

**Methods:**

Pathological tissue samples were taken during surgery on patients with primary and recurrent pterygium. Healthy conjunctive tissue samples were taken during cataract surgery. After homogenization of 77 tissue samples, evaluation was made of thiobarbituric reactive substances (TBARS), nitric oxide (NO), total antioxidant status (TAS) and the activity of the three main antioxidant enzymes: glutathione peroxidase, superoxide dismutase and catalase. Gender differences were evaluated.

**Results:**

Compared to the control group, in the primary pterygium group there was an increase in NO and TAS, and a tendency to a decrease of all antioxidant enzymes, indicating an increase in non-enzymatic antioxidant activity. Compared to the control group, in the recurrent pterygium group there was a significant decrease in the level of TAS and antioxidant enzymes. A high positive correlation was found between most of measured parameters within the control group and the recurrent pterygium group, but not within the primary pterygium group. Compared to men, a significant difference was observed in the elevated NO level and low TAS level of women in the prymary pterygium group.

**Conclusions:**

The diminished antioxidant defense in the recurrent pterygium group, possibly determined mainly by decreased non-enzymatic activity, supports the idea that oxidative stress plays an important role in the recurrence of this disorder.

## Background

Pterygium is an inflammatory and degenerative process resulting from an uncontrollable cellular proliferation of the subconjunctival and fibrovascular connecting tissue on the cornea. Chronic exposure to ultraviolet light, causing the excessive production of free radicals through a photochemical reaction, is widely accepted as an important factor in the development of pterygium [1, 2]. This disease causes changes in the oxidant/antioxidant state of the human cornea [3, 4]. The molecular mechanism by which tissue proliferation is induced is still not clear. In a recent revision [5] a mutagenic mechanism, with the participation of ROS generated by UV light, was discussed as the principal trigger of non-cancer ocular diseases including pterygium. On the other hand, a return of disease after the operation may be due to a non-mutagenic mechanism. This possibility requires further research that compares primary and recurrent pterigium. Data about these differences were analyzed in a review [6], which explored the role of the p53 tumor suppressor [7, 8], the different growth factors [9] and distinct viruses [10, 11].

In the context of primary pterygium, there are various reports on oxidative stress and the antioxidant defense [12–18]. Elevated levels of nitric oxide (NO) and reduced levels of SOD and CAT have been found in the tissue of primary pterygium, which suggests the involvement of oxidative stress in this pathology [15, 16]. 8-hidroxideoxiguanosine (8-OHdG), a sensitive and stable marker commonly used to identify oxidative damage to DNA, has been found in pterygium in some studies [12, 13, 18, 19]. Nevertheless, no relation has been established between 8-OHdG and recurrent pterygium [18]. Indeed the information on the oxidant/antioxidant state is scarce in regard to this disorder.

In rabbit, it has been shown that consumption of antioxidants as a nutritional supplement can strengthen defenses against oxidative stress involved in cornea and conjunctiva [1]. In humans this same therapy is used to treat marginal dry eye [20]. The aim of the present study was to compare the oxidant/antioxidant state of tissue from primary and recurrent pterigium in men and women of a population exposed to UV light during many days of the year.

## Methods

There were 92 patients involved in the present two-year study, including those with healthy tissue (C), primary pterygium (PP) and recurrent pterygium (RP). Exclusion factors were the existence of systemic illness, immunosuppressive treatment, or previous ocular surgery. The same group of doctors operated on the patients with pterygium and cataracts. Based on pre-established criteria, 15 patients were excluded from the study, leaving 77 participants. In the case of recurring patients, the time between the first and second operation varied between 2 and 8 years. Regarding gender and age differences among patients in the three groups, there were no significant differences (Table 1).

**Table 1:** **Population data**

Group	n	Age (male)	n	Age (female)	n	Age (all)
Control	12	60.2 ± 8.4	11	54.1 ± 17.5	23	56.6 ± 17.0
Primary pterygium	16	55.5 ± 8.6	15	54.0 ± 8.5	31	54.7 ± 9.1
Recurrent pterygium	11	47.6 ± 14.5	12	53.6 ± 10.2	23	50.7 ± 13.1

Gender and age of patients in the control, primary pterygium and recurrent pterygium groups.

Values are given as the mean ± SD. n = number of patients.

The study was conducted in the Superior Medicine School of the National Polytechnic Institute, and a hospital known as Nuestra Señora de la Luz. Both institutions are in Mexico City (altitude 2300 m). The pathological tissue samples were obtained during surgery on patients with primary and recurrent pterygium. Samples of healthy conjunctive tissue were obtained during cataract surgery from the nasal limbus area. All tissue samples were collected with the patient’s consent. The protocol was approved by the Ethics Committees of the Superior Medicine School based on criteria’s that adhered to the tenets of the Declaration of Helsinki.

Samples were immediately placed in liquid nitrogen to await homogenization (within 4 weeks). Homogenization of samples was carried out in cold phosphate buffer (30 mmol/l, pH 7.4, 0.1% Triton ×100), followed by centrifuging (10,000 rev/min, 15 minutes at 4°C). The supernatant was stored at -70°C to await analysis. Total proteins were determined within 2 weeks of homogenization by employing Lowry’s method. Measurement was made of thiobarbituric acid reactive substances (TBARS) [21], nitrates/nitrites (NO) and catalase (CAT) by using Caymanchemistry procedures. The Randox procedure was used to determine the total antioxidant status (TAS), as well as glutathione peroxidase (GPx) and superoxide dismutase (SOD) levels. Results are presented in nmol/mg of total protein for TBARS, NO and TAS, and in U/mg of total protein for the enzymes.

TAS was measured in the system that generates the ABTS® cation radical (HX-Fe^III^ + H_2_O_2_), with absorbance at 600 nm. In the event of the presence of antioxidants in stained tissue, absorbance is diminished. A synthetic antioxidant (6-hydroxy-2,5,7,8-tetramethylchroman-2-carboxylic acid) was used as the standard. Thus, the level of TAS is equivalent to nmol/mg of total proteins of this standard.

The software package SPSS 17.0 was used for all statistical analysis. One-way ANOVA (one way) and the Tukey test were used to analyze variables between groups, and the Pearson correlation coefficient was employed for values within each group. Statistical significance was considered with p < 0.05.

## Results and discussion

**Figure 1:**
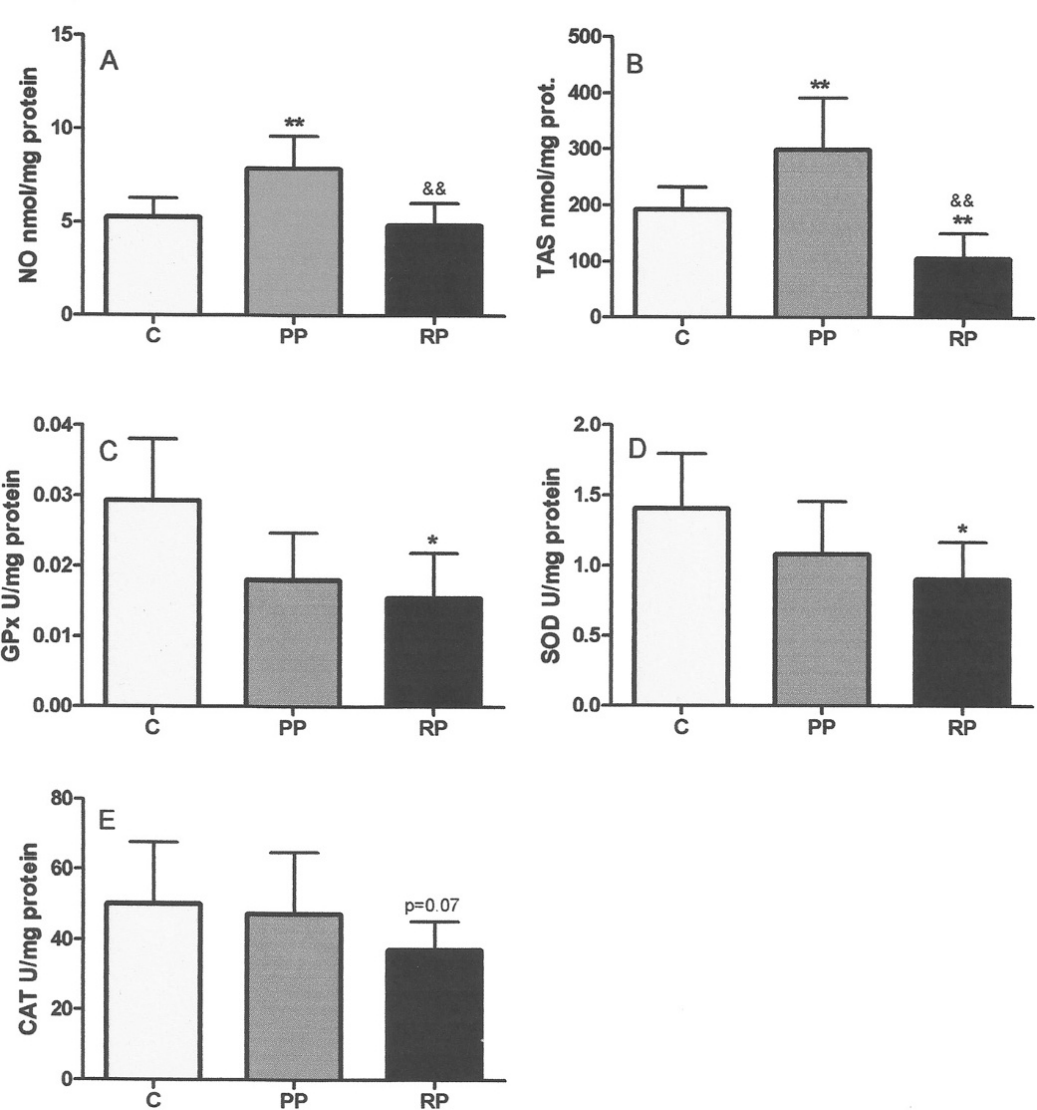
**All patients.** Levels of NO **(A)**, TAS **(B)**, GPx **(C)**, SOD **(D)** and CAT **(E)** in the three groups: control group (C); the primary pterygium group (PP); and the recurrent pterygium group (RP). n = number of patients. * - p < 0.05, ** - p < 0.01 compared to the control group. & - p < 0.05, & & - p < 0.01 compared PP and RP groups.

There were no statistical differences in the TBARS levels between any of the groups (all patients). The level of NO (Figure 1A) and TAS (Figure 1B) of the primary pterygium group was significantly higher than the value of these parameters for the control or recurrent pterygium groups. The level of TAS in the recurrent pterygium group was lower than that in the control group. For the three principal antioxidant enzymes (GPx, SOD and CAT), there was a tendency to lower levels in the primary pterygium than control group (Figure 1C, D, and E, respectively), and significantly lower levels in the recurrent pterygium than control group.

**Figure 2:**
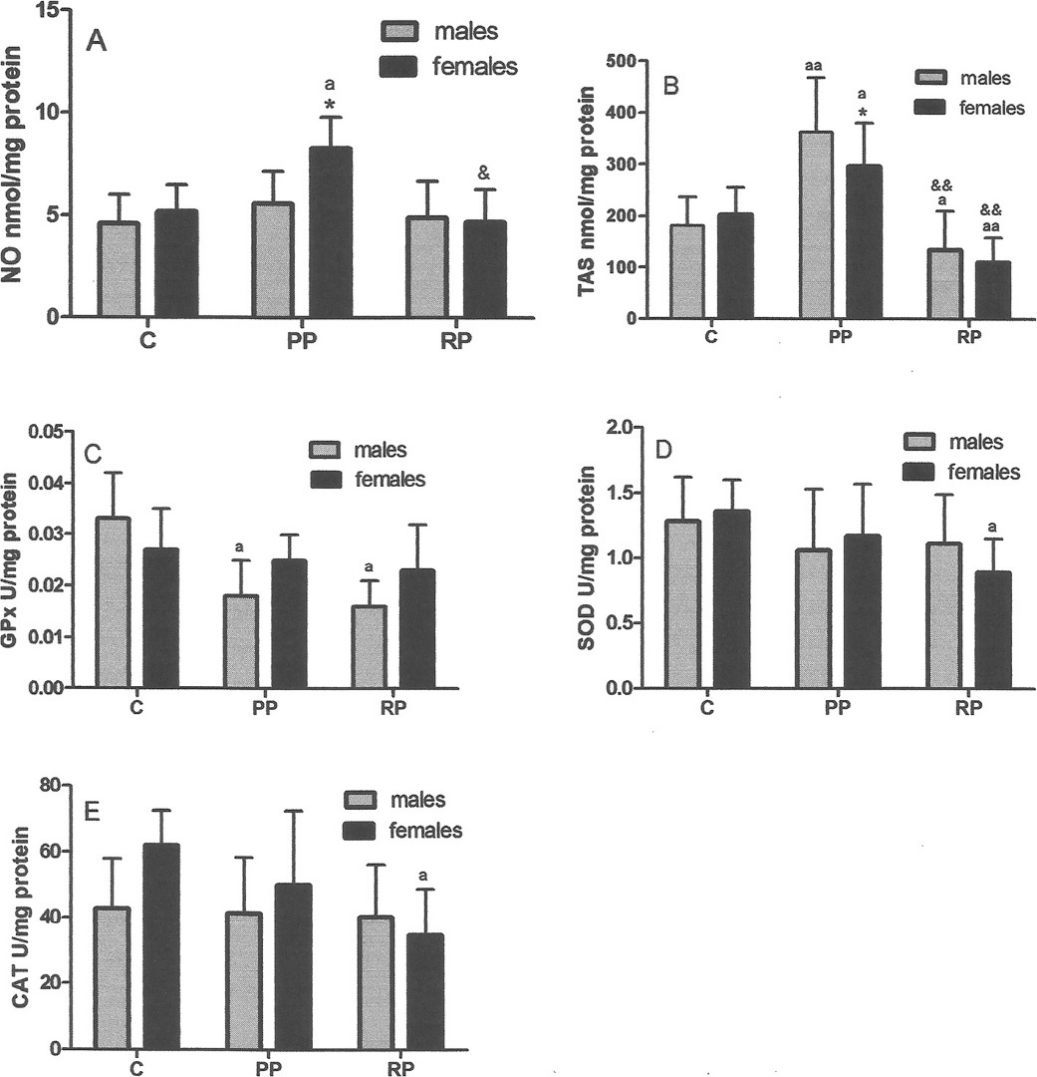
**Genders differences.** Levels of NO **(A)**, TAS **(B)**, GPx **(C)**, SOD **(D)** and CAT **(E)**, in the three groups: the control group (C), the primary pterygium group (PP) and recurrent pterygium group (RP). * - p < 0.05, ** - p < 0.01 regarding gender differences within each group; ^a^ - p < 0.05, ^aa^ - p < 0. 01 compared to the same sex of the control group; & - p < 0.05, && - p < 0.01 when comparing the same sex between the PP and RP groups; m = male; f = female.

Regarding TBARS levels, there was no significant difference between groups or between genders within each group. A significant difference existed in NO levels between men and women of the primary pterygium group, and between women of this same group and the women of the other two groups (Figure 2A). That is, the higher average level of NO found in all patients of the primary pterygium was caused principally by the women of this group. There was a higher level of TAS for both men and women in the primary pterygium group than in the corresponding gender of the control group (Figure 2B). Contrarily, there was a lower level of TAS for both men and women in the recurrent pterygium group than in the corresponding gender of the control or PP group. Regarding enzymatic activity (Figure 2C, D, E), no gender difference was observed within any group. However, in regard to men was a significantly lower value of GPx in the PP and RP groups compared to the control group (Figure 2C). Additionally, in regard to women the value of SOD and CAT was lower in the recurrent pterygium group than the control group (Figure 2D, E).

It is commonly accepted that a close relationship exists between the parameters of oxidative stress and those of the antioxidant defense. We calculated the Pearson correlation coefficients between the values of parameters within each group. A correlation was not observed between two parameters of oxidative stress (TBARS and NO) in all groups. When comparing the values of TBARS, NO and antioxidant parameters inside of groups, a correlation was found in the control and recurrent pterygium group, but not in primary pterygium group (Table 2). There seem to be some factors that distort and/or transform this relation in latter group. A gender difference was observed principally in the control group, where SOD activity had a positive correlation with all parameters for women, but only with GPx for men. For men and women of the primary pterygium group, the SOD activity only corresponded positively to the value of CAT. For men and women of the recurrent pterygium group, the SOD activity showed a positive correlation with all parameters of the antioxidant defense. Additionally, for men of this same group, the SOD activity showed a positive correlation with parameter of oxidative stress. It seems that the high level of correlation of SOD with other parameters in patients of control group was mainly caused by the women of this group.

**Table 2:** **Correlation between parameters**

	Group	n	TBARS	NO	TAS	GPX	CAT
SOD (all)	C	23		0.54*	0.51*	0.71**	0.69**
	PP	31					0.87***
	RP	23		0.55*	0.85***	0.47*	0.86***
SOD (f)	C	11		0.86**	0.62*	0.77*	0.72*
	PP	15					0.88**
	RP	12			0.83**	0.82**	0.85**
SOD (m)	C	12				0.69*	
	PP	16					0.95**
	RP	11	0.79*	0.69*	0.86**		0.87**
CAT (all)	C	23		0.79***			
	PP	31					
	RP	23		0.48*	0.87***	0.59*	
CAT (f)	C	11		0.90**		0.72*	
	PP	15					
	RP	12			0.87**	0.60*	
CAT (m)	C	12					
	PP	16				0.65*	
	RP	11	0.67*		0.62*		
GPx (all)	C	23					
	PP	31			0.59*		
	RP	23					
GPx (f)	C	11		0.64*			
	PP	15			0.84**		
	RP	12			0.68*		
GPx (m)	C	12					
	PP	16					
	RP	11					

Matrix of the Pearson correlation coefficients between measured parameters of the oxidant/antioxidant state in ocular tissue of all patients, female and male within each group: control (C), primary pterygium (PP) and recurrent pterygium (RP). * - p < 0.05, ** - p < 0.01, *** - p <0.001; no value – p > 0.05. n = number of patients per group, m = males, f = females.

SOD = superoxide dismutase, CAT = catalase, GPx = glutathione peroxidase, TAS = total antioxidant status, TBARS = thiobarbituric acid reactive substances. NO = nitric oxide.

CAT response shows the same SOD pattern in the distribution of correlations for the groups under study, both in all the patients and in those separated by gender. The GPx correlations were observed in the PP group in all patients and females. No correlations were observed between indicators of oxidant stress (NO, TBARS), and between TAS with these indicators, except for NO in the RP group of males.

The elevated level of NO in the primary pterygium group compared to the control group coincides with the data of various reports [14–16, 22], although it is not in agreement with one study [14]. This controversy was discussed in a recent study [16]. There was a clear tendency to lower levels of the three principal antioxidant enzymes in the primary pterygium group compared to the control group (without showing statistical significance), as was found in the aforementioned repot [16]. Unlike that study, in the current contribution no significant difference was found in the level of TBARS of the primary pterygium group compared to the control group.

The higher level of NO in the primary pterygium group (compared to the control group) coincides with the elevated level of TAS and with the tendency to lower levels for all three antioxidant enzymes in the same group. For various reasons we interpret the level of TAS as the sum of enzymatic and non-enzymatic antioxidant capacity: (i) in the homogenized tissue, both enzymatic and non-enzymatic antioxidant activity is present; (ii) the possibility exists for stable free radicals (used in the methodological procedure) to be inactivated by both non-enzymatic and enzymatic antioxidants, as all antioxidant enzymes represent metalloproteins; (iii) the positive correlation of this parameter with the levels of all antioxidant enzymes in this study provides indirect evidence in favor of this interpretation. Although our interpretation is debatable, we will use it as long as there is no contrary evidence.

Thus compared to the control group, the higher level of TAS together with the lower level of the principal enzymatic antioxidant activity in the primary pterygium group implies a higher level of non-enzymatic antioxidant activity in this same group. Compared to the control group, there was no significant difference in the parameters of oxidative stress (TBARS and NO) in the recurrent pterygium group, but the values of the parameters of the antioxidant defense (TAS and the three enzymes) were significantly lower in this same group. The decrease is similar among these four parameters, varying between 30 to 40%. As there is no evidence of a decrease in the generation of ROS in this group, we can conclude that the system of antioxidant defense in recurrent pterygium group is strongly debilitated, at least in the enzymatic part, compared to the control group. When comparing the recurrent pterygium group with the primary pterygium group, only NO and TAS were found to be significantly lower in the former, representing 40% and 68% of the values of the latter group, respectively. Considering that the decrease in enzymatic activity in the recurrent group compared to the primary group is less than 15% (p > 0.1), it can be appreciated that the differences between these two groups are determined mainly by non-enzymatic part of TAS activity. If true, the patients of the recurrent pterygium group have a deficient non-enzymatic part of antioxidant defense compared with patients of the primary pterygium group.

Overall, the similarity in the correlations between the different parameters in the control group and the recurrent pterygium group (Table 2) is quite notable, as is the difference between these two groups and the primary pterygium group. It is likely that greater non-enzymatic antioxidant activity is the main factor leading to the distinct results for the latter group. Non-enzymatic antioxidants can be mobilized to ocular tissues from other organs (including the liver). For some reason the level of vitamin C in the subconjunctival connecting tissue of the cornea is around 1 mmol/l, higher than in any other tissue in the human organism [3]. Whereas it is likely that patients with recurrent pterygium have already exhausted this source of antioxidants, patients with primary pterygium probably still have this source available.

Regarding gender differences, a significantly higher level of NO was found in the women than men of the primary pterygium group. The increase in NO levels in the current contribution was smaller than that found in a recent report [16], probably because women comprised 65% of pterygium group in that study and only 50% in our study. The elevated level of NO could indicate a greater level of oxidative stress, and is likely to be important for the integral functioning of the antioxidant defense by NO mediated mechanism [23].

The gender difference in correlation between parameters of antioxidant defense was observed principally in the control group. SOD activity had a positive correlation with all parameters of the antioxidant defense for the women of this group, but showed a positive correlation only with the value of GPx for the men. This confirms that gender differences do indeed seem to exist in the functioning of the antioxidant system. In the presence of oxidative stress in normal conjunctive tissue, it seems that the non-enzymatic antioxidant capacity plays a greater role in men than women with regard to the maintenance of oxidant/antioxidant homeostasis.

Whether the cause is the depletion of the non-enzymatic antioxidant reserves (due to poor nutrition, poor functioning of the digestive tract or some other reason) or a higher degree of oxidative stress, the result is the same— a weakening of the antioxidant system and consequently an increase in the risk of a recurrence of pterygium. Additionally, it cannot be ruled out that the weakening of the antioxidant defense in the recurrent pterygium group is due to a reduced genetic expression of the main antioxidant enzymes, especially in people with little physical activity.

## Conclusions

The existence of a diminished antioxidant defense in the recurrent pterygium group supports the idea that oxidative stress plays an important role in the return of pterygium. It is possible that the differences between two pathological groups are determined mainly by non-enzymatic antioxidant active, suggesting the importance of maintaining the antioxidant defense of patients after surgery of primary pterygium [5, 24].

## Abbreviations

TAS: Total antioxidant status
TBARS: Thiobarbituric acid reactive substances
NO: Nitric oxide
GPx: Glutathione peroxidase total
SOD: Superoxide dismutase total
CAT: Catalase.
